# Assessment of Postresuscitation Volume Status by Bioimpedance Analysis in Patients with Sepsis in the Intensive Care Unit: A Pilot Observational Study

**DOI:** 10.1155/2016/8671742

**Published:** 2016-08-15

**Authors:** Bram Rochwerg, Jason H. Cheung, Christine M. Ribic, Faraz Lalji, France J. Clarke, Susheel Gantareddy, Nischal Ranganath, Aziz Walele, Ellen McDonald, Maureen O. Meade, Deborah J. Cook, Trevor T. Wilkieson, Catherine M. Clase, Peter J. Margetts, Azim S. Gangji

**Affiliations:** ^1^Department of Medicine, McMaster University, Hamilton, ON, Canada; ^2^Department of Clinical Epidemiology and Biostatistics, McMaster University, Hamilton, ON, Canada; ^3^Division of Nephrology, St. Joseph's Healthcare Hamilton, Hamilton, ON, Canada; ^4^Department of Family Medicine, Northern Ontario School of Medicine, Sudbury, ON, Canada; ^5^Brampton Civic Hospital, Brampton, ON, Canada

## Abstract

*Background*. Bioimpedance analysis (BIA) is a novel method of assessing a patient's volume status.* Objective*. We sought to determine the feasibility of using vector length (VL), derived from bioimpedance analysis (BIA), in the assessment of postresuscitation volume status in intensive care unit (ICU) patients with sepsis.* Method*. This was a prospective observational single-center study. Our primary outcome was feasibility. Secondary clinical outcomes included ventilator status and acute kidney injury. Proof of concept was sought by correlating baseline VL measurements with other known measures of volume status.* Results*. BIA was feasible to perform in the ICU. We screened 655 patients, identified 78 eligible patients, and approached 64 for consent. We enrolled 60 patients (consent rate of 93.8%) over 12 months. For each 50-unit increase in VL, there was an associated 22% increase in the probability of not requiring invasive mechanical ventilation (IMV) (*p* = 0.13). Baseline VL correlated with other measures of volume expansion including serum pro-BNP levels, peripheral edema, and central venous pressure (CVP).* Conclusion*. It is feasible to use BIA to predict postresuscitation volume status and patient-important outcomes in septic ICU patients.* Trial Registration*. This trial is registered with clinicaltrials.gov NCT01379404 registered on June 7, 2011.

## 1. Introduction

Severe sepsis confers an extremely high mortality rate of approximately 40% in critically ill patients [1]. Early goal directed therapy (EGDT) with aggressive fluid resuscitation has been shown to reduce mortality by 16% in a single-center randomized trial [2]. Two subsequent multicenter trials found no clear benefit to EGDT; however, patients randomized to both protocolized and nonprotocolized care in both of these studies received early and aggressive fluid resuscitation [3, 4]. Beyond the initial resuscitative period, observational studies have identified that a persistent hypervolemic state has been independently associated with mortality, prolonged mechanical ventilation, and need for renal replacement therapy [5–7]. Although aggressive fluid resuscitation remains the cornerstone of EGDT, subsequent management of fluid status in patients with severe sepsis has not been well defined. An added challenge is that accurate volume assessment in patients in the intensive care unit (ICU) is difficult to ascertain.

Subjective assessment of volume status by physical examination is limited by poor accuracy and reliability [8]. Other objective measures such as central venous pressure and pulmonary catheter pressure are invasive and not predictive of volume status [9–12]; therefore, tools to help guide clinicians in assessing patients' fluid status are required. Bioimpedance analysis (BIA) assesses the electrical properties of tissues by measuring the reactance and resistance of an alternating current passed through the body. BIA values can be plotted on reactance-resistance (*R*-*X*_*c*_) graphs to derive a vector length (VL). The VL is a reflection of an individual's overall volume status. A longer VL is indicative of euvolemia or volume depletion and a shorter VL is associated with volume expansion [13]. In patients with end-stage renal disease on dialysis, VL has been correlated inversely with volume status [14–18]. In patients with congestive heart failure, BIA has been shown to predict volume status; in these patients, VL was shown to increase with diuresis, suggesting that VL is a reflection of overall volume [19]. In addition, BIA devices are practical tools to use at the bedside, being compact, portable, inexpensive, and noninvasive.

BIA has not been well studied in the ICU setting. In a single-center cross-sectional study comprising 34 critically ill medical and surgical patients [20], VL was found to correlate with central venous pressure (CVP) measures (*r* = −0.38, *p* = 0.025). However, the use of BIA to assess total volume status and prognosis in a prospective manner in critically ill patients with sepsis has not been reported. We performed a prospective observational pilot study to determine the feasibility of using BIA in the ICU. Our pathophysiologic hypothesis was that septic patients would have an initial period of volume expansion (reflected in low VL values), eventually resolving during the first few days of their ICU admission (as seen by increasing VL values) as fluid was mobilized and the patient moved towards no longer requiring invasive mechanical ventilation (IMV). Feasibility measures included recruitment efficiency [21], eligibility and enrolment, and consent rate. Our secondary objectives were to determine if VL was associated with the need for IMV and other clinical measures of volume, thereby assessing the construct validity of VL as a marker of volume.

## 2. Methods

This prospective single-center study was conducted from January 17, 2011–Feb 1, 2012. Inclusion criteria were adult patients (age > 18 years) admitted to the ICU with systemic inflammatory response syndrome (SIRS) and a high clinical suspicion of infection, requiring invasive positive pressure ventilation, and with a central venous catheter (internal jugular or subclavian) allowing CVP measurement. Exclusion criteria were preexisting end-stage kidney disease on chronic dialysis, pregnancy, limb amputation(s), the presence of a temporary or permanent pacemaker, and inability to obtain informed consent. The study received approval from the Hamilton Integrated Research Ethics Board (HIREB) prior to patient enrolment. All patients who participated in this study provided explicit informed consent either directly or via their substitute decision maker.

Baseline data collected at enrolment included age, sex, race, height, weight, CVP (cm H_2_O), APACHE II score, heart rate (HR), mean arterial pressure (MAP), urine output (mL/hr), multiple organ dysfunction score (MODS), and need for life support modalities. Enrolled patients were classified as medical or surgical (having received a surgical procedure within 72 hours of ICU admission). A seven-point Likert scale assessing peripheral edema was created and performed in duplicate for 45 of the measurements in order to evaluate reliability (see Supplementary Material available online at http://dx.doi.org/10.1155/2016/8671742 for edema scale). These duplicate measures were performed independently and the evaluators were blinded to one another's assessment. We used the intraclass correlation coefficient (ICC) to estimate agreement on the duplicate peripheral edema scores (*r* = +1 represents perfect agreement; *r* = 0 represents no agreement). Laboratory data collected at baseline included routine ICU blood work in addition to N-terminal brain natriuretic peptide (N-BNP) levels.

BIA measurements were performed at enrolment (within 48–96 hours of ICU admission), and then at day 3 and day 7 after enrolment using the Bodystat Quadscan 4000 (Bodystat, Isle of Man, British Isles). Patients were assessed in the supine position on nonconductive surfaces. Tetrapolar placement of disposable foil-gum electrodes (wrist-hand and ankle-foot) was used. Measurements were performed at 5, 50, 100, and 200 Hz and done in triplicate with the average of these three measures used for analysis (see Supplementary Material for an example of an individual patient's bioimpedance readings with means and standard deviations). Bioimpedance standard operating procedures were developed for this study and distributed to all study sites to ensure consistency of technique (see Supplementary Material). Also, face-to-face training was provided to all research staff involved in performing measurements that included direct observation. Transformation of the data was completed according to the Piccoli method [22]. The first 28 patients enrolled had a total of three BIA measurements performed within the first week after enrolment. BIA VL data for 8 of these initial patients was lost due to human and/or device error. After analysis of the temporal changes in BIA in these first 20 patients, we concluded that more frequent BIA measurements conducted over an extended period of time would be required in order to demonstrate proof of concept. Consequently, a protocol change was instituted for the remaining patients such that BIA measurements were performed at baseline and on day 3 and day 7 and every four days until day 30 (or ICU discharge or ICU death). If patients were discharged from ICU prior to day 30, they were followed on the ward with a single subsequent BIA measurement. Physicians responsible for the clinical care of the recruited patients, nurses, study investigators, and the research coordinator were blinded to the BIA test results.

Our primary outcome for this pilot was the feasibility of performing BIA vector length measures in the ICU setting. The initial study proposal focused on efficacy outcomes; however, given the protocol changes that were required after the first 20 patients were enrolled, we changed our primary focus to feasibility. Feasibility determination included recruitment efficiency, eligibility and enrolment, and consent rate. Secondarily, our proof of concept determination involved evaluating whether a correlation existed between initial BIA vector length and other measures of volume status, both intra- and extravascular including CVP, N-BNP serum levels, edema score, cumulative fluid balance, and total volume of fluid infused. Cumulative fluid balance and total volume of fluid infused were reported throughout the study period. Other patient-important outcomes included determining if an association existed between VL and ventilator status (i.e., having a status of requiring IMV versus not requiring IMV) and the proportion of patients who developed acute kidney injury as determined by the RIFLE criteria. Stage risk (RIFLE-R) and stage failure (RIFLE-F) were evaluated. RIFLE-R is defined as 1.5–2x increase in serum creatinine from baseline, a 25% decrease in estimated glomerular filtration rate (eGFR), or a urine output < 0.5 mL/kg/hr for any consecutive 6-hour period. RIFLE-F is defined as 3x increase in serum creatinine from baseline, a 75% decrease in eGFR, or a urine output <0.3 mL/kg/hr for any consecutive 24-hour period or anuria.

Baseline BIA measurement data points for each patient were correlated with other baseline measures of volume status using the Pearson statistic. We used the ICC statistic to estimate agreement on the duplicate peripheral edema scores. A generalized estimating equation (GEE) was used to determine if a relationship between VL and ventilator status and VL and proportion of patients meeting RIFLE-R or RIFLE-F criteria was present. The GEE allowed us to estimate the parameters in our generalized linear model with adjustment for potential correlation between variables with multiple repeated measures. Potential confounders controlled for in the GEE included age, vector length, and study day. Statistical significance was set at *p* < 0.05.

## 3. Results

**Figure 1:**
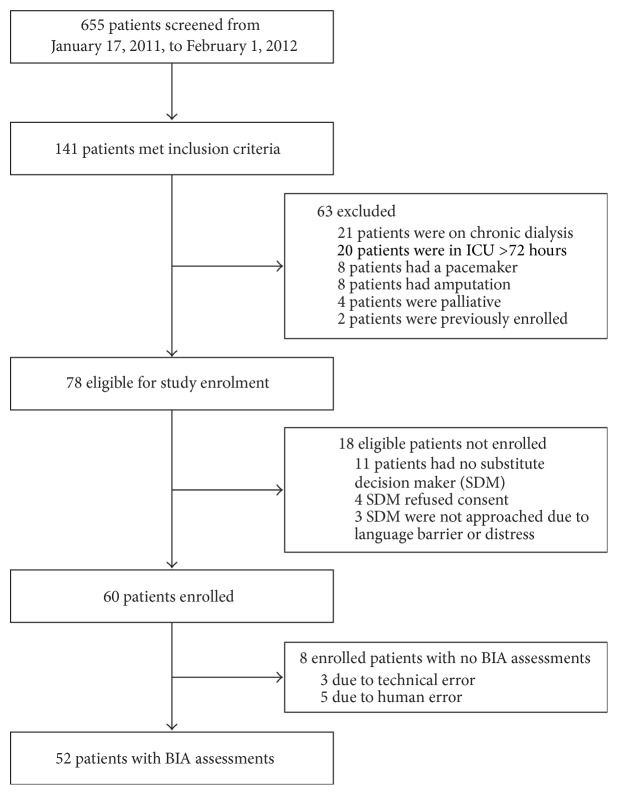
Flowchart of patient enrolment.

A total of 655 patients were screened (Figure 1). Of these, 141 patients (21.5%) met the inclusion criteria, of which 63 patients subsequently met exclusion criteria. A further 18 eligible patients were not enrolled due to consent refusal (four patients), no substitution decision maker available (eleven patients), or language barrier/family distress (three patients). Therefore, 60 patients were enrolled (with a consent rate of 93.8%) over the 12-month recruitment period. Due to device malfunction (3 patients) and human error (5 patients), the BIA measurement values were not available for 8 patients. The average monthly recruitment rate was 6.15 patients enrolled per month. There were no reported patient complications from BIA measurement acquisition. Each measurement required less than 10 minutes to perform.

**Figure 2:**
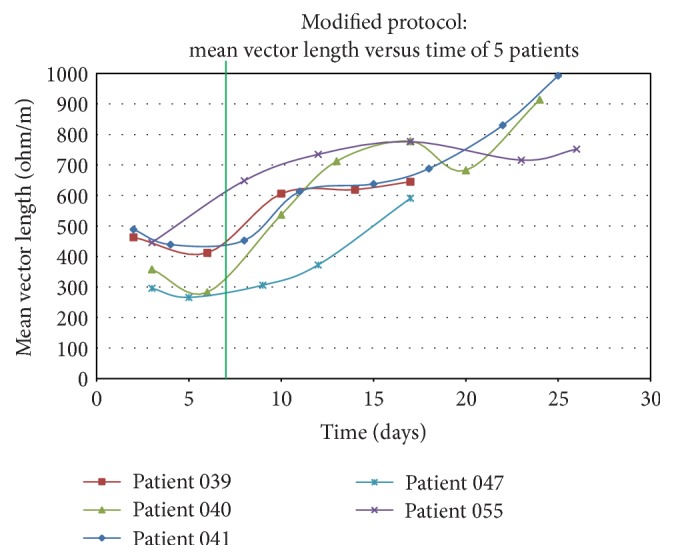
Change in vector length over time. Change in mean vector length over time is shown for 5 randomly selected patients. The green vertical line is shown at day 7 and demarcates the initial 7-day protocol (followed for the first 16 patients) from the subsequent 30-day protocol. Persistently small vector lengths (representing hypervolemia) are seen for the first 7 days which led us to increasing the number of measurements and length of follow-up for this feasibility study. There is significant variability within each patient's BIA measures, suggesting more frequent measures are required to more accurately assess volume status over time.

Our study protocol was altered after we reviewed data from the first 20 patients. The initial protocol called for three BIA measures over the first week after enrolment. Initial measures suggested patients remained volume overloaded at day 7 with little change in their VL measurements (Figure 2). We therefore decided to expand the study period to better capture the change in volume status over time. After the amended protocol was approved by the HIREB, the BIA measurements were performed at baseline and on day 3 and day 7 and every four days until day 30 (or ICU discharge or ICU death). Thirty-two patients had up to 8 BIA assessments during their ICU stay. Even while performing BIA measurements every four days, variability among individual patient's data points was still significant (Figure 2).

A total of 52 patients completed the study with a mean APACHE II score of 26.8 (SD 6.9). Baseline characteristics are presented in Table 1. Considering correlation with other known measures of volume status, shorter baseline VL measurements (suggesting volume overload) were associated with higher CVP readings (*r* = −0.21, *p* = 0.03), higher N-BNP levels (*r* = 0.302, *p* = 0.04), and higher scores on our edema scale (*r* = −0.673, *p* < 0.001) (Table 2). Agreement for our edema score was excellent (*r* = 0.73). Correlations with cumulative fluid balance, total volume of fluid infused, and serum albumin levels were not significantly associated with VL.

For each individual 50-unit increase in VL (ohm/m), there was an associated 22% increase in the probability of not requiring IMV. This was not statistically significant (*p* = 0.13). Similarly, for each 50-unit increase in VL (ohm/m), there was a 32% decrease in meeting RIFLE-R criteria (*p* = 0.06) and a 65% decrease in meeting RIFLE-F criteria (*p* = 0.07).

**Figure 3:**
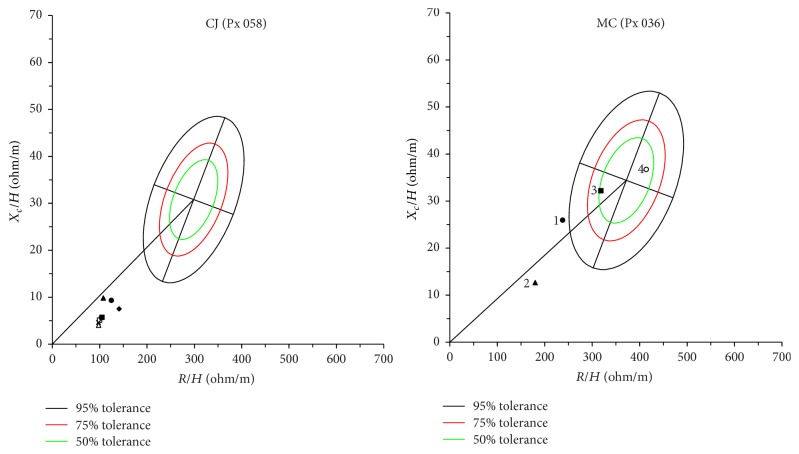
*R*-*X*_*c*_ (resistance versus reactance) graphs of two individual patients. An illustrative example of *R*-*X*_*c*_ graphs demonstrating the volume trajectories of two enrolled patients. Patient 58 had 8 measurements done while in the ICU and was never extubated. It is evident that all of the measurements for patient 58 remain in the left lower quadrant of the *R*-*X*_*c*_ graph suggesting a persistently small vector length (or a hypervolemic state). Patient 36 had 4 measurements done while in the ICU and was extubated after the 2nd measurement. Patient 36 started in the hypervolemia range; however, the patient moved to the upper right quadrant of the *R*-*X*_*c*_ graph consistent with improved volume status and extubation (numbered dots indicate visit day).

Figure 3 shows an example of contrasting clinical trajectories plotted on *R*-*X*_*c*_ graphs for two individual patients enrolled in our study. The lower left quadrant of the graph represents volume overload and shorter vector lengths while the upper right quadrant represents hypovolemia and longer vector lengths. Patient 58 represents a patient who remained volume overloaded throughout their ICU stay and was never successfully extubated. Patient 36 had VL measurements that increased over time and the patient was extubated on study day twelve.

## 4. Discussion

This was a prospective observational cohort study designed to examine BIA as a tool to measure overall postresuscitation volume status in ICU patients. Current measures of volume status are inaccurate and at times rely on invasive procedures. A novel method of determining postresuscitation volume status is needed and BIA represents an inexpensive, noninvasive, and reliable alternative to our current state of practice. Although our initial focus in this trial was to assess efficacy, the need for early protocol modifications led us to feasibility targets with a secondary objective to study the impact on patient-important outcomes and the correlation of BIA measurements with other known measures of volume status. Through this pilot, we have demonstrated the feasibility and that the BIA technique is an easily implementable tool in the ICU setting. Although measurements for 8 patients were lost, this was due to administrative error and battery failure rather than a problem with the BIA machine itself. Our enrolment rate (percentage enrolled compared to those screened) was 8% with fourteen patients missed due to inability to contact a substitute decision maker. If we had included these fourteen patients in the study, as would be possible using an alternate consent model such as deferred consent, and assuming consent had been obtained, our enrolment rate would have been at our target of 10%. Our consent rate for this feasibility study was excellent (93.75%), meeting our target of greater than 80%.

This pilot study was not powered to evaluate clinically relevant patient outcomes. Despite this, we observed two trends (neither reached statistical significance): patients with longer VL (less hypervolemia) may be less likely to require IMV (*p* = 0.13) and may be less likely to develop acute kidney injury based on RIFLE criteria (*p* = 0.07). This latter finding is consistent with the observational data [5–7, 23, 24] and limited RCT data [25] that suggest a link between ongoing hypervolemia and renal dysfunction. VL was significantly and inversely associated with other markers of volume expansion, CVP, and edema score. This provides some criterion validity with respect to VL as a measure of total volume in critically ill patients. No association between VL and cumulative fluid balance or total fluid infused was evident; however, these measures are often unreliably and inconsistently recorded in the ICU [26].

Previous clinical applications of BIA have relied on regression-based equations which make assumptions based on healthy populations and lack validation in critically ill patients [27]. By using the Piccoli method [13], and *R*-*X*_*c*_ graphs comparing resistance against reactance, we have been able to circumvent these issues. Only one other prospective observational study [20] has investigated the use of BIA in thirty-four ICU patients with a predominant admitting diagnosis of trauma. Those enrolled had serial BNP, CVP, and BIA measurements performed throughout their ICU stay. Baseline measurements showed a weak correlation between CVP and BIA VL (*p* = 0.025); however, this did not persist beyond the initial measurement. Also, there was no correlation between BIA VL and oxygenation index. Importantly, this study did not focus on postresuscitative patients with sepsis or examine the effect on patient-important outcomes such as ventilator requirements.

Our original hypothesis suggested that septic patients would have an initial period of volume expansion that would resolve within the first few days after ICU admission. Based on initial results, we found that patients were significantly volume expanded at time of study enrolment (two days after ICU admission) and remained persistently volume overloaded even up to a week after admission. As previously mentioned, we therefore modified our protocol and began performing additional BIA measurements every four days for up to 30 days (or ICU discharge or ICU death). This observed delay in fluid mobilization in postresuscitation septic patients was significant and may warrant further investigation. The clinical importance of “deresuscitation” is increasingly being recognized and BIA could be used as a tool to help guide this process.

Strengths of this feasibility study include the novel application of a relatively inexpensive and easy-to-use, bedside device to help address the question of postresuscitation volume status in critically ill patients. Our study design enabled real-time protocol enhancements and improved data collection. We focused on a specific patient population and excluded patients who were admitted to ICU and received volume resuscitation for etiologies other than sepsis. Correlating the BIA VL measurements with other known intra- and extravascular measures of volume status allowed for proof of concept assessment. Other strengths include assessing the reliability testing of the edema score.

Limitations of this study include the relatively small number of patients who were all enrolled from a single ICU. Given the pilot nature of our study, it is difficult to draw any conclusions about clinically important outcomes based on these data. Despite the suggestion that BIA may be a predictor of ventilator status [28], there are many factors in addition to volume status that are associated with ventilator status in critically ill patients. Significant within-patient variability of BIA VL was noticed with the measurements being performed every four days and, therefore, in the future, consideration will be given to performing daily measurements to better capture the true variation in this measure.

## 5. Conclusions

Clinicians faced with the challenge of managing hypervolemia beyond the acute resuscitative phase of critical illness lack a noninvasive and reliable method to guide volume management. Based on this pilot, BIA measures are significantly associated with traditional measures of resuscitative volume status such as CVP and BNP and postresuscitation hypervolemia such as edema score thereby adding to its validity as a measure of volume. Bioimpedance analysis may have a role in the management of sepsis beyond the initial resuscitative phase with regard to volume assessment and management, including aiding in reducing the associated untoward consequences. The results of this feasibility study have fueled the protocol of a large observational study that is currently underway to determine whether BIA vector length can predict patient-important outcomes such as ventilator dependence and AKI.

## Supplementary Material

This supplementary material provides the 7-point Likert scale that was used to assess peripheral edema in patients enrolled in this study.

## Figures and Tables

**Table 1:** Baseline patient characteristics at time of study enrolment.

Characteristic	*N* = 52
Age (years), mean (SD)	64 (13)

Sex	
Male, *N* (%)	27 (52%)

Ethnicity	
Caucasian, *N* (%)	47 (90%)
Black, *N* (%)	3 (6%)
Aboriginal, *N* (%)	1 (2%)
Other, *N* (%)	1 (2%)

Weight (kg), mean (SD)	82.4 (27.6)

Patient type	
Medical, *N* (%)	46 (89%)
Surgical, *N* (%)	6 (11%)

Chronic health index, median (min, max)	1 (0–2)

APACHE II score, mean (SD)	26.8 (6.9)

MODS score, mean (SD)	7.5 (2.9)

Vasopressor/inotropic dependence on admission, *N* (%)	40 (77%)

Serum creatinine on admission to ICU *μ*mol/L, mean (SD)	143.4 (127.7)

**Table 2:** Correlation of baseline vector length with other baseline measures of volume status.

Clinical feature	Pearson correlation coefficient (*R*)	*p* value
N-BNP serum level	−0.30	0.04
Central venous pressure	−0.21	0.03
Edema score	−0.67	<0.001
Cumulative fluid balance	−0.22	0.11
Volume of fluid infused since admittance (crystalloid & colloid)	−0.19	0.18
Albumin serum level	0.271	0.079

## Abbreviations

BIA: Bioimpedance analysis
ICU: Intensive care unit
VL: Vector length
RIFLE: Risk, injury, failure, loss, end stage
BNP: Brain natriuretic peptide
CVP: Central venous pressure
EGDT: Early goal directed therapy
*R*-*X*_*c*_ graph: Resistance versus reactance graph
IMV: Invasive mechanical ventilation
SIRS: Systemic inflammatory response syndrome
HIREB: Hamilton Integrated Research Ethics Board
HR: Heart rate
MAP: Mean arterial pressure
APACHE: Acute physiology and chronic health evaluation
MODS: Multiple organ dysfunction score
ICC: Intraclass correlation coefficient
Hz: Hertz
eGFR: Estimated glomerular filtration rate
GEE: Generalized estimating equation
RCT: Randomized controlled trial
AKI: Acute kidney injury.
