# Supplementary material for: Assessment of Postresuscitation Volume Status by Bioimpedance Analysis in Patients with Sepsis in the Intensive Care Unit: A Pilot Observational Study
Source: Can Respir J. 2016 Aug 15;2016:8671742. doi: 10.1155/2016/8671742 (PMC5002474; doi:10.1155/2016/8671742)
Supplement: Supplementary file 1 — This supplementary material provides the 7-point Likert scale that was used to assess peripheral edema in patients enrolled in this study. [file 8671742.f1.pdf]

Appendix I. 7-point edema score.

1	2	3	4	5	6	7
No Edema	Trace	Slight Pitting	Obvious swelling	Swelling with Deep Pitting	Severe Diffuse Swelling	Anasarca

Appendix II. Example of Bioimpedance Readings from Individual Patient

Patient #32 – BIA measurements

Time Point	Measurement	Resistance	Mean (SD)
1	1	330.17	
	2	329.75	
	3	329.54	329.82 (0.32)
2	1	333.81	
	2	334.75	
	3	333.8	334.12 (0.55)
3	1	316.77	
	2	317.78	
	3	317.74	317.43 (0.57)
4	1	416.71	
	2	416.73	
	3	416.71	416.72 (0.01)

Appendix III. Bioimpedance Standard Operating Procedure (SOP).

This SOP outlines the procedure for bioimpedance and related measurements.

1. Ensure subject:
   - has four limbs.
   - does not have any electrical and battery operated implantable devices (i.e. Pacemaker, cochlear implants, insulin pump implants).
   - is not on dialysis (at the point of recruitment into bioimpedance substudy).
   - is not pregnant.
2. Weigh the subject (kg).
3. Ensure the subject is supine in the ICU bed.
4. Turn on the bioimpedance device by pressing the ON/OFF button . Record the device number. Record the test number on the subject case report form (CRF), enter the subject gender, age and weight.
5. While subject is lying still, use alcohol wipes to clean areas and place electrodes as follows:
   a. RED CABLES → below 2nd and 3rd knuckles on the hand, and below 2nd and 3rd toes
   b. BLACK CABLES→ on the wrist beside the ulnar head and on the ankle between the lateral and medial malleoli (the bones on the sides of the ankle) Notes: It may be necessary to shave the site of excess hair to prevent poor contact. **It is crucial for the ankle and wrist electrodes to be placed correctly to ensure accurate measurements.**

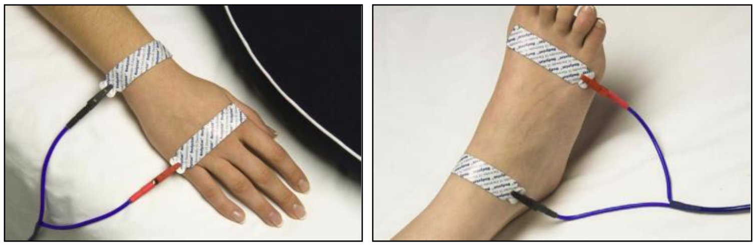

Source: BODYSTAT® QuadScan 4000 Hardware User's Guide 2007

6. Connect cables to electrodes, ensuring that the metal contact is against the foil side of the electrode and that the black and red cables are connected correctly. Once in place, press down on the electrodes to ensure good contact.
7. Start measurement by pressing the enter key on the bio-impedance device.

8. Cycle through the output with the ↵ key until you reach the resistance 50kHz and reactance 50kHz values. Record both of these values on the CRF. If these values are passed by in error, record “missed” on CRF as these data is stored electronically in the machine. Note: Based on previous data, typical values for resistance 250Ω-700Ω and reactance 12Ω-75Ω.

9. Repeat test two more times. Record all three test numbers on the CRF. Record all three sets of resistance and reactance values.

10. Remove the electrodes.

11. Thank the subject (if applicable) and the bedside nurse.
